# Design guidelines for movement-assistive clothing based on a comprehensive understanding of older adults’ needs and preferences

**DOI:** 10.1371/journal.pone.0299434

**Published:** 2024-03-20

**Authors:** Jiwon Chung, Wujun Tang, Jeong Eun Yoon, Suhyun Ha, Ju Young Kang, Sumin Helen Koo

**Affiliations:** 1 Department of Clothing and Textiles, Yonsei University, Seoul, South Korea; 2 Department of Fashion Design and Merchandising, University of Hawaii at Mānoa, Honolulu, Hawaii, United States of America; Sunway University, MALAYSIA

## Abstract

This study aimed to explore the needs and wants of older adults in the context of movement-assistive clothing (MSC), with a focus on muscle strength and posture correction. A survey was conducted to understand the needs and wants of older adults, considering aspects of functions and designs, and to evaluate the comfort, safety, ease of use, usefulness, and intention of users to purchase and use products. A total of 408 individuals aged > 65 years participated in the study. The data were analyzed using descriptive analyses, such as mean, standard deviation, percentages, Cronbach’s alpha, chi-square test, independent *t*-test, analysis of variance, and regression using IBM SPSS 27.0. Exploratory Factor Analysis was also conducted to test the hypotheses. Open-ended questions were extracted using major themes after color-coding. Based on the results, design recommendations were derived, including the development of pants and innerwear with casual, minimalist styles, featuring achromatic colors, and utilizing stretchy, breathable fabrics. Comfort, safety, ease of use, and usefulness emerged as critical factors influencing the purchase and use of MSC by older adults. This study aimed to establish design guidelines by understanding the needs and wants of older adults and considering the aspects of movement-assistive clothing to relieve musculoskeletal issues. Accordingly, these findings are expected to aid in the creation of wearable suits using flexible fabric artificial muscles for active musculoskeletal correction in older adults.

## Introduction

As the population ages and workforce shrinks [1], there is an increase in musculoskeletal issues [2] and costs for rehabilitation and chronic disease care [3]. Older adults often face musculoskeletal issues due to decreased muscle and bone densities [2]. Deformities of the waist, legs, neck, and shoulders can lead to chronic pain, physical abnormalities, paralysis, and limitations in performing daily activities. As the digital generation commonly has musculoskeletal issues such as turtleneck syndrome [4], these problems are expected to become more widespread. Therefore, we would need to prepare for an aged society and help people who are or may have difficulties moving and who suffer from musculoskeletal issues.

Functional clothing for musculoskeletal issues, such as compression aids or waistbands, is passive. For more active functions, wearable robots, such as exoskeletons, have been developed to address musculoskeletal challenges from aging and for greater independence, improved quality of life, and cost efficiency. However, passive aids can cause swelling symptoms owing to compression with little support. Exoskeleton robots composed of heavy and rigid materials are unsuitable for daily wear, causing strain, unnatural movements, and potential injuries over time. Therefore, soft wearable robots have been developed that offer potential benefits such as comfort owing to their light weight, flexibility, and ease of use [5,6].

Among soft wearable robots, clothing-integrated robots are considered a key advancement, outperforming traditional robots in terms of compatibility and affordability [6,7]. These clothing-integrated types would need to address core clothing factors, such as wearability, durability, and maintenance. Many products use embedded components, such as sensors and actuators, for complicated tasks, such as washing. Requiring users to wear the same item daily or multiple robot products also reduces their appeal. To create a successful product that blends clothing and ensures user-friendliness, it is necessary to systematically address these core clothing requirements and understand the wearer’s needs and wants. However, there is a gap in user-focused research, which has led to limited commercial products. Early design phases should emphasize the essential usage requirements. In addition, most existing wearable suit designs do not consider the needs and desires of older adults [5–7]. A wearable suit design would also need to cater to the movement assistance needs of older adults and align them with the user needs and wants.

Therefore, this study aimed to understand the needs and wants of older adults and consider aspects of movement-assistive clothing (MSC) to relieve musculoskeletal issues. Based on the results, design guidelines were established. These findings are expected to aid the creation of wearable suits using flexible fabric artificial muscles for active musculoskeletal correction in older adults. This research can pave the way for innovative technologies, offer an international competitive edge, and boost commercialization potential.

## Literature review

### Changes in aging and clothing requirements

It is vital to understand age-related body changes when developing MSC for older adults. Aging affects body composition, causing various physical and chemical alterations, such as changes in the skin, musculoskeletal system, fat and body shape, vision and hand agility, and thermoregulation. These patterns emphasize the need for age-adapted clothing designs.

First, proteins such as collagen decrease skin elasticity as people age [8], affecting flexibility and movement. Aging skin has fewer nerve endings, which leads to reduced sensitivity. Flat seams minimize irritation in older adults, while external stitching reduces skin contact [9]. Methods such as fewer seams or layered designs improve comfort and thickness concerns [9]. Strategies include the use of soft fabrics for seam ends and the adjustment of materials in the friction areas for greater comfort. Additionally, the skin becomes thinner, more prone to wrinkles, and has less sebum. Decreased melanocyte numbers make the skin more sensitive to ultraviolet rays [10,11], thereby increasing the risk of UV damage. Therefore, older adult friendly clothing would need to offer flexibility, comfort in movement, and thermal properties without reducing skin sensitivity. Clothing can be made with soft and smooth materials such as organic cotton, lyocell, Tencel, and modal, provide UV protection, and have fewer seams and stitches; however, if they exist, they are made of flat seams and external stitches.

Second, older adults experience changes in their musculoskeletal systems, including reduced bone density, muscle strength, and muscle mass. Muscle strength decreases by 1–1.5% annually from ages 50 to 70 and drops even faster afterward [11], and studies indicate that muscle mass and strength peak decline accelerates after age 70, resulting in a 15% decrease in muscle mass and a 25–40% decrease in strength every ten years [12]. As people age, their joints become stiffer and tire more quickly because of decreased tendon elasticity and weaker connections among the musculoskeletal components, increasing the risk of injury and slowing recovery [10]. Aging also affects the cartilage cushioning capability [11], leading to increased bone-on-bone friction. Changes in the lower body include pelvic tilt, widened stance, and lowered center of gravity [13,14]. These musculoskeletal changes highlight the importance of clothing design in the older adult population. Thus, MSC is necessary for older adults and can be developed separately for those older than 70. Major functions include muscle strength assistance for better movements and/or correct joint posture to reduce injury risk and enhance recovery.

Third, fat distribution changes with age, leading to body shapes such as shorter height, thinner limbs, thicker waists, longer neck and back with reduced front-center length, and more fat on the slouched shoulders and rounded back. The waist and abdominal measurements increase, making the abdomen more circular. Body shape changes accentuate in the 80s and intensify post-85, and there are also gender differences. Older women show more varied body changes than men [14,15]. For older women, there are decreases in breast circumference, increases in thicker necks that are on average 2 cm longer than those in the 20s, and in neck-nipple length, affected by age-related sagging, with a 4 cm increase than that of women in their 20s, and reduced curvature with minimal chest-waist differences and a hip flatness ratio of 0.75 [16–18]. These data highlight the need for design adjustments to accommodate distinct body contours. Conversely, older men show chest reduction and decreased leg circumferences in the thighs and claves by 2–3 cm compared to people in their 20s and decreased back fat and upper arm circumferences compared to their middle-aged peers [18,19]. Scapular thickness increases, and increased shoulder inclination results in more rounded shoulders (right,23.30°; left,22.85) [20]. Therefore, it is recommended to develop patterns of MSC by decreasing the total lengths of the clothing and the front-center length but adding more lengths to the back neck and back, less ease allowances on limbs, and more ease allowances on the abdominal, waist, shoulder, and back circumferences. Considering gender differences, for older women, the ease of breast circumference, flatter hips, and increased total length of the front bodice are suggested. For older men, decreased body circumference, including the chest and back, decreased upper arm circumference, but increased shoulder circumference.

Fourth, aging impedes dressing and routine tasks due to a decline in vision and hand agility, affecting movement and joint flexibility [12,14]. A decline in neurotransmitter release and central nervous system function also slows the muscle response by 10–15% during activities [21]. As age advances, physical capabilities wane, necessitating clothing designs that emphasize comfort and ease of wear with easy fastening and slits.

Older adults experience compromised thermoregulation, decreased temperature sensitivity, and weakened homeostasis. Reduced sweat glands and blood vessels affect heat regulation and increase the risk of heatstroke in hot weather [10]. This diminishes their ability to regulate the temperature, making cold adaptation vital. In warmer settings, delayed bodily responses and elevated core temperatures affect heat tolerance. Additionally, decreased sweat production disrupts thermal regulation, resulting in skin dryness [9,22]. To address the decreased temperature regulation in older adults, fabric treatments for moisture absorption and temperature control should be considered. Fibers with strong capillary action, such as wicking fabrics, absorb water effectively, allow rapid evaporation, and enhance comfort [23]. The utilization of such materials increases the comfort and satisfaction of older adults for better temperature regulation. Extant research on MSC development has not considered older adults’ needs, wants, and requirements [5–7]. Hence, the defined requirements can guide the development of survey questionnaires, design guidelines, and MSCs to relieve musculoskeletal issues in older adults.

### Considering aspects and use/purchase intention

The design of wearable systems necessitates a thorough consideration of various factors, including safety, ergonomics, comfort, and reliability. Human wearability involves several crucial design considerations, including safety, stability, comfort, and overall ease of wear [24]. The Technology Acceptance Model (TAM) has been applied to understand the acceptance level and use intention of new technologies [25–27] and for studies on older adults [28,29]. However, there is a lack of research on MSCs in older adults [25–31]. Thus, it would be beneficial to identify whether TAM is acceptable and whether the related requirements are likewise applicable when developing MSCs for older adults. According to the TAM, perceived usefulness and ease of use are significantly correlated with the intention to use [25]. In addition to perceived usefulness and ease of use, the perceived comfort [26,30,31] and safety [26,32] can affect the purchase intention of products like MSC. It would be helpful to investigate how users’ perceptions of usefulness, ease of use, comfort, and safety affect their intention to use and purchase MSCs. The results can be applied to decide which aspects should be focused on when developing MSCs.

### Hypotheses

**Fig 1:**
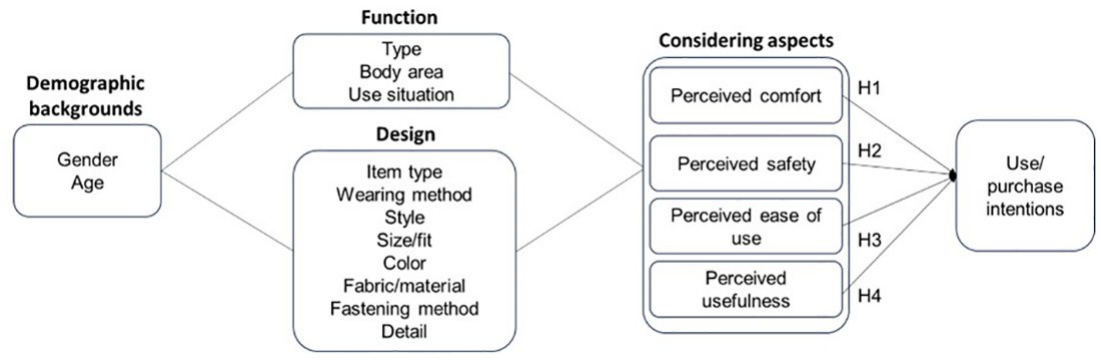
The research model on MSCs.

Analyzing the physiological reactions of the human body after wearing clothing is meaningful in terms of clothing comfort [30]. Comfort can be described as a physical or psychological sensation, and it is crucial to consider user’s comfort [31]. The perceived comfort of a wearer significantly affects the usability of wearable technologies [31]. Perceived comfort in smart clothing has been found to significantly influence the perceived ease of use [26], which affects the purchase intention. Therefore, if MSC is more comfortable, people will be more willing to use and purchase them (Fig 1).

*H1*. Higher perceived comfort of MSCs increases use or purchase intentions of MSCs.

With the increasing sophistication and ubiquity of robots, the importance of safety becomes increasingly important [32]. To be considered safe, specific guidelines must be followed. While the utilization of soft robots undeniably offers numerous advantages, it is essential to be cautious about the potentially misleading assertion of inherent safety [32]. Perceived risk negatively influences purchase intention for smart clothing [26]. For older adults, if they have more technology anxiety, they show less positive attitudes toward technology [33]. If products are perceived as high-risk, they may not want to use or purchase them.

*H2*. Higher perceived safety of MSCs increases use or purchase intentions of MSCs.

Another factor indicating the level of ease of use in the user’s perception is the perceived ease of use [34]. Perceived ease of use refers to the degree to which individuals believe that using a product decreases their effort [25]. It is an individual’s perception that it requires minimal effort. When individuals perceive that using technology demands significant exertion, they are less likely to intend to use it [35]. Users are more likely to accept an application if they find it easier to use than others [25]. The ease of use of smart clothing influences purchase intentions [26]. Considering these factors, the perceived ease of use may influence levels of use and purchase intention.

*H3*. Higher perceived ease of use increases the intention to use or purchase MSCs.

Perceived usefulness refers to the degree to which individuals believe that using a product improves their job performance [25]. Perceived usefulness is an individual’s conviction about the efficacy of a particular system, indicating that people believe technology can facilitate the achievement of their objectives [35]. When making purchasing decisions, users typically opt for products or services that offer the highest perceived value, and purchase intention serves as a gauge of the likelihood that a user will make a purchase [36]. When individuals recognize the benefits associated with technology usage, their inclination to employ it increases [35]. User behavioral intentions are influenced by perceived usefulness [37]. Therefore, the perceived usefulness can affect the purchase intention of a product.

*H4*. Higher perceived usefulness of MSCs increases use or purchase intentions of MSCs.

## Materials and methods

After obtaining IRB approval from XX University (No. 7001988-202307-HR-1975-02), participants over 65 years of age, recognized as older adults [38], and computer literate were selected. Those willing clicked ‘agree’ to start the online survey, and the informed consent form was exempted. The online survey ran from July 25 to August 2, 2023.

To understand older adults’ needs and wants, considering aspects and their relationships with the use and purchase intention of MSCs, a survey was conducted with older adults. The survey questionnaire was borrowed from the existing literature [14,30,35,39–44] and referred to literature reviews on changes in aging and clothing requirements, considering aspects and use/purchase intentions with the TAM model to test the hypotheses.

The survey had 48 multiple-choice and rating scale questions (1 = strongly disagree, 7 = strongly agree): seven on demographics, five on needs and wants in function (e.g., function, assistive body parts, corrective body parts, use situation, other suggestions) [39–41], nine on needs and wants in design (e.g., item type, wearing method, style, fit or size, color, fabric, fastening method, detail, other suggestions) [40,42,43], and 23 on considering for using and purchasing MSC (e.g., 9Q comfort, 6Q safety, 4Q ease of use, 4Q usefulness) [30,35,41,42,44], three on use and purchase intention [14,40,41], and one open-ended question for the suggestion.

The data were analyzed using descriptive statistics, Cronbach’s alpha, chi-square, independent *t*-test, one-way analysis of variance (ANOVA), regression in IBM SPSS 27.0, and Exploratory Factor Analysis (EFA). The open-ended questions were theme-coded. Based on the literature and a survey, design guidelines for muscle-assistive clothing for older adults were created. The Cronbach’s alpha values ranged from .894 to .956, indicating high internal consistency.

## Results and discussion

### Demographic backgrounds

A total of 408 older adults, consisting of 204 men and 204 women, participated in the study. The majority were in their 60s (66.4%), followed by those in their 70s (27.2%), and 80s (6.4%). Occupationally, 32.8% of the respondents were retired or unemployed, 31.9% were housekeepers, and 9.8% were experts. More than half of the participants reported being healthy (50.5%), while others rated their health as average (29.9%), unhealthy (13.5%), very healthy (4.7%), or very unhealthy (1.5%).

### Needs and wants in functions and designs

**Fig 2:**
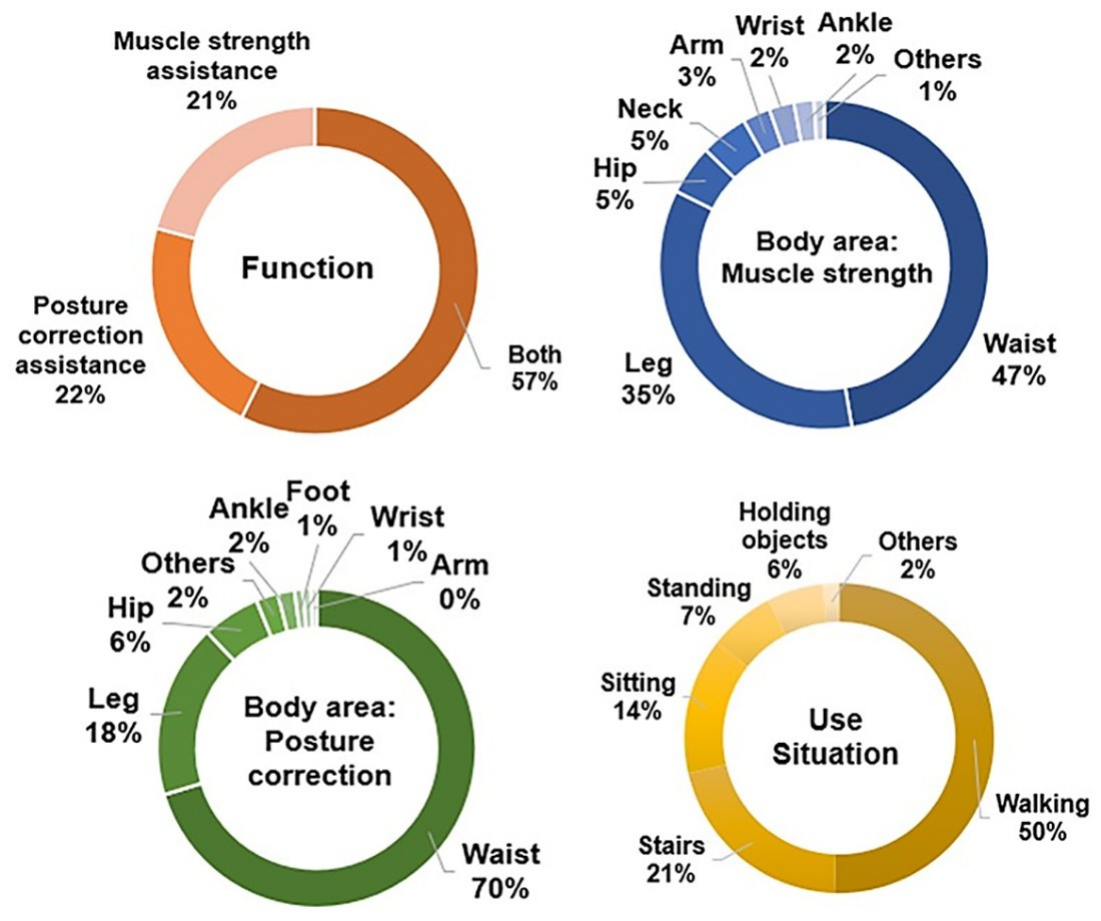
The needs and wants of older adults in functions for MSCs.

Over half of the participants (57.4%) desired both muscle strength assistance and postural correction from MSCs (Fig 2). The preferred areas for both muscle strength and posture correction assistance were the waist and legs. More individuals preferred posture correction assistance for the waist (61.50%) than muscle strength assistance (47.3%). Other desired features included vital signals, such as electrocardiogram and blood pressure, monitoring musculoskeletal health conditions, losing weight, and relieving pain. Waist assistance was needed and desired owing to cartilage calcification, and muscle mass decreases with age [9,11,12,40]. Aging also causes major leg changes such as pelvic tilting, leg spreading, and knee bending [13,14]. Most participants preferred clothing aids for walking (50.50%), followed by stairs (20.80%) and sitting (14.20%). Hence, the development of MSCs for the waist and legs during activities, such as walking, ascending or descending stairs, and sitting, is recommended.

**Fig 3:**
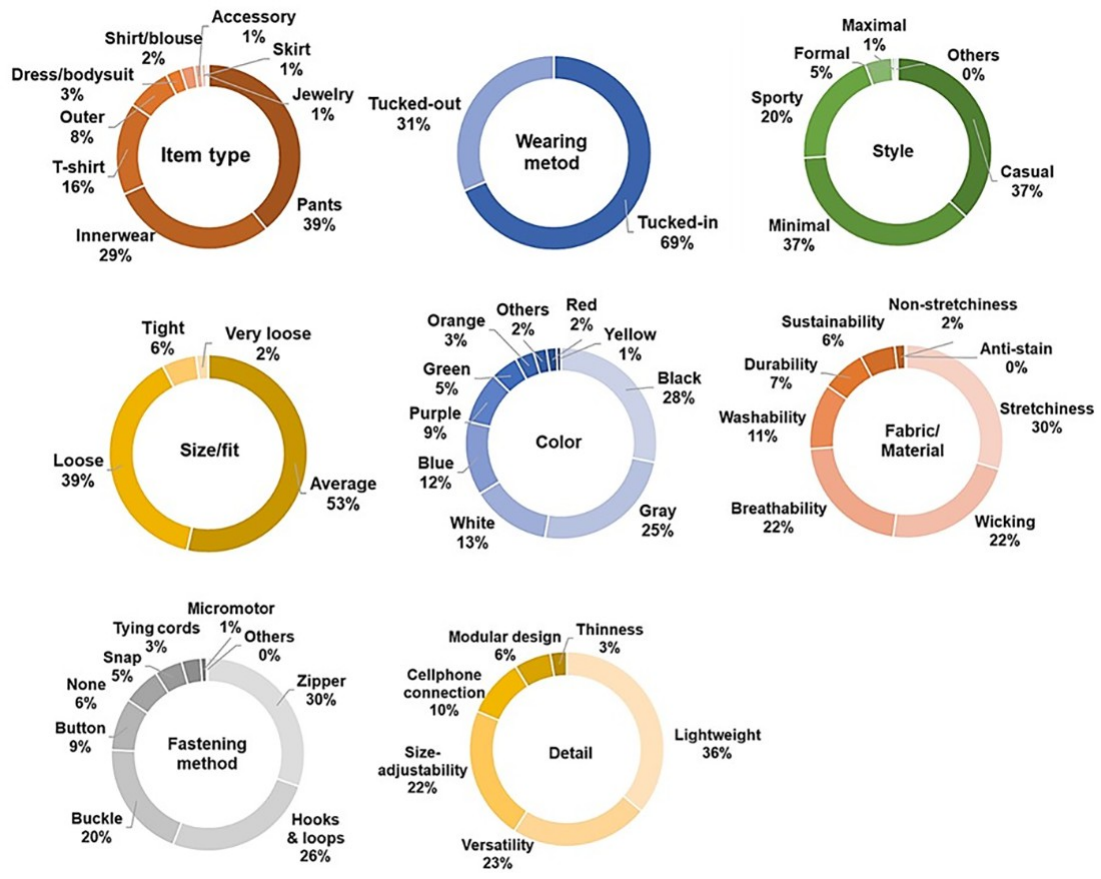
The needs and wants of older adults in designs for MSCs.

In terms of design, pants (39.20%) were the most desired, followed by innerwear (29.20%) and t-shirts (16.20%) (Fig 3). People prioritize features such as stretchiness, comfort, wearability, light weight, reasonable price, washability, and non-stickiness without causing skin problems. More people (68.60%) wanted to wear inside their clothing, which is why the inner-wear type was highly desired. Casual and minimal styles were the most popular at 37.00% each, and over half (53.40%) preferred the average size or fit.

Black (28.20%) was the top color choice, followed by gray (24.50%) and white (12.50%), which are all achromatic colors. This aligns with previous research indicating a preference for achromatic colors, especially black, to avoid drawing unnecessary attention [45–47]. People preferred fabrics with stretchiness (29.70%), wicking (22.50%), and breathability (21.60%) for comfort in terms of movement and thermal aspects. Zippers (30.40%) and hooks and loops (25.50%) were the top closure choices, which made the MSC wear similar to existing normal clothing. In terms of details, lightweight (36.00%) was the most desired, followed by interchangeable (23.00%) and size-adjustability (22.30%). People suggested design features such as length adjustability, pockets, front zippers, no skin residue, suitability for outdoor or sportswear, and shareability with the family. Specifically, lightweight was the most desired, followed by versatility and size adjustability. Other desired features can also be incorporated to satisfy different user needs.

### Considering aspects and use/purchase intention

**Fig 4:**
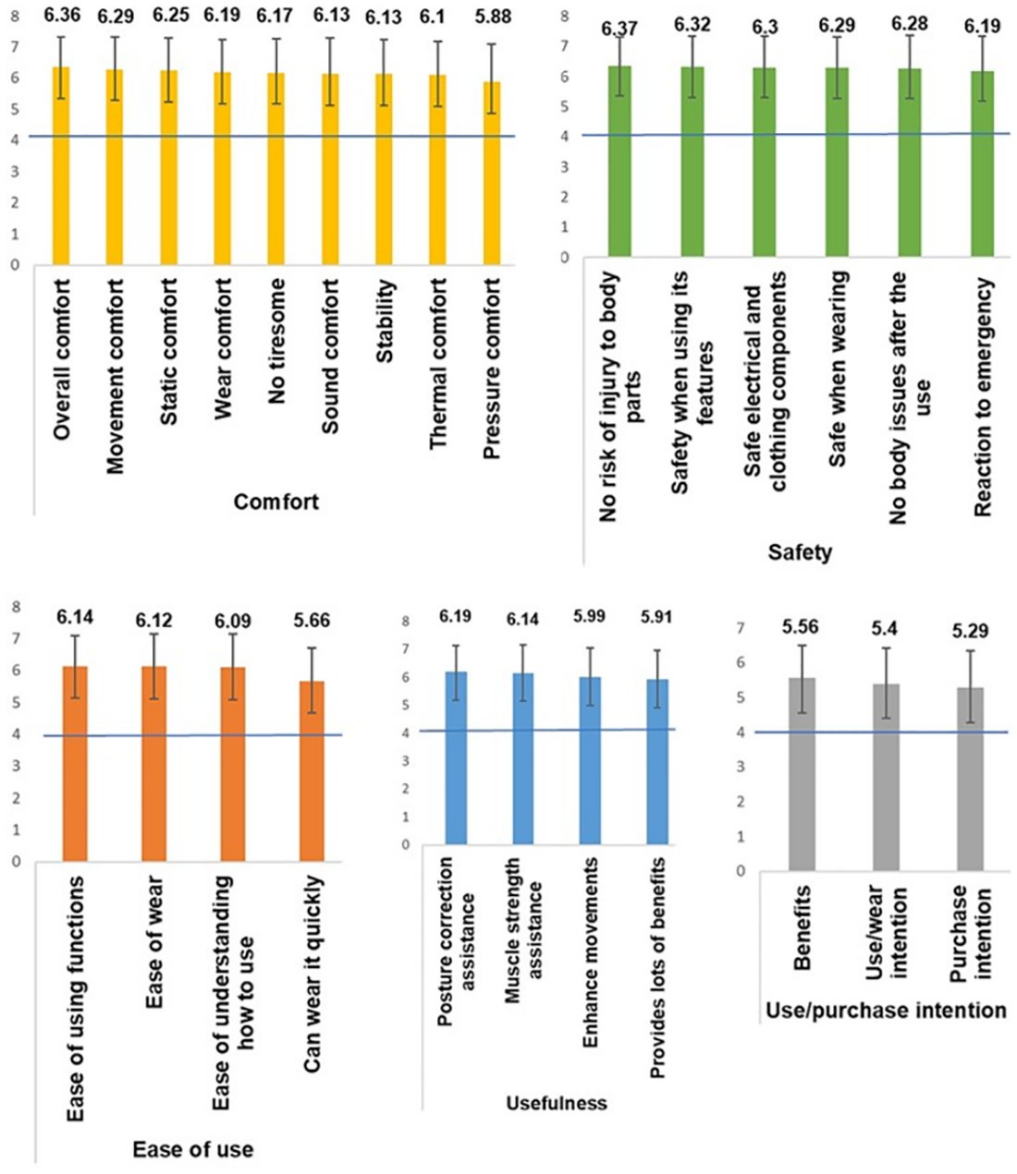
Considering aspects when purchasing MSCs.

When purchasing MSCs, comfort, safety, ease of use, and usefulness were considered important (5.66 < M < 6.37) (Fig 4). Safety scored highest, especially “no risk of injury to body parts by equipment” (M = 6.37, SD = 1.02). Over 96.8% of the responses scored above 4 on the Likert scale, indicating that older adults prioritized safety. Comfort (M = 6.36, SD = 0.95) followed closely, and all comfort questions scored above 94.4%, indicating that older adults highly valued comfort in MSCs. Usefulness peaked for posture correction assistance (M = 6.19, SD = 1.07), with all questions scoring above 95.6%. Ease of use (M = 6.14, SD = 1.07) scored the highest, and all questions surpassed 95.1%. Compared to safety and comfort, ease of use and usefulness were slightly lower. However, with an average score above 5.66 and consideration rates exceeding 95.1%, older adults still deem ease of use and usefulness crucial when purchasing MSCs. Therefore, developers would need to satisfy wear comfort, injury prevention, functional safety, ease of operation, and functions such as posture correction and muscle strength assistance.

Approximately 90.4%–95.8% of respondents expressed interest in using and purchasing MSCs. The intention to use and wear was slightly higher than that to purchase, but both averaged over 5.29, indicating the older adults’ willingness toward these MSCs. They believed these products would offer benefits (M = 5.56, SD = 1.21). Thus, MSCs are deemed necessary and appealing for older adults.

### Differences in gender

**Fig 5:**
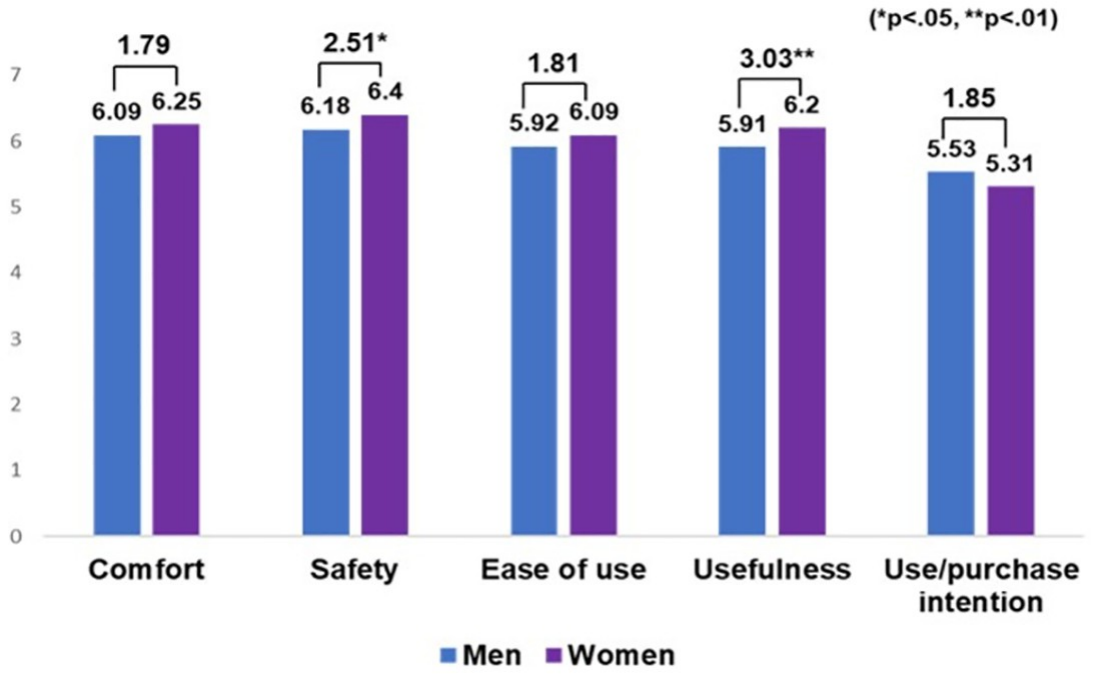
Results of *t*-tests of gender (mean, *t*-value, and *p*-value).

Gender-based independent *t*-tests revealed significant differences in how older men and women perceived MSCs. Women rated safety (t = 2.51, *p* < .05) and usefulness (t = 3.03, *p* < .01) more highly than men (Fig 5). This aligns with past studies showing that older women undergo more body changes than men [14,15], hence they emphasized safety and usefulness. When designing MSCs for older women, emphasis on safety and usefulness is necessary. However, both genders viewed comfort, safety, ease of use, and usefulness as critical and showed a strong intention to use and purchase MSCs.

**Fig 6:**
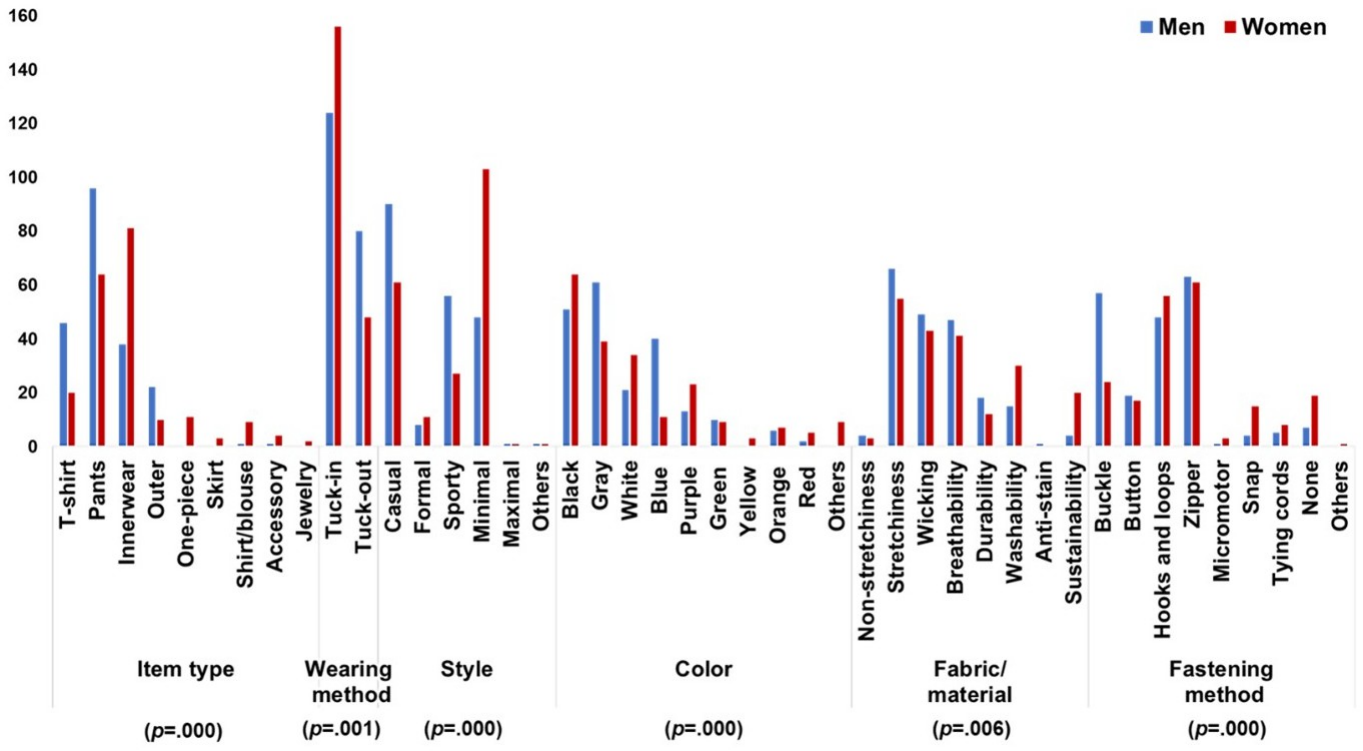
Results of chi-square test of gender.

Based on the two-tailed chi-square test (expected frequency < 5; sample size > 40), there were no significant gender differences in function. However, the designs showed significant differences between genders in all aspects (*p* < .05) except for size or fit and detail (Fig 6). The men’s top three items were pants, t-shirts, and innerwear. Women preferred innerwear, pants, t-shirt, plus one piece, skirts, and accessories, and more wanted to wear inside of the clothing than men. Men’s style preferences were casual, sporty, and minimal, whereas women’s style preferences were minimal, casual, and sporty. Men favored gray, black, and blue colors, whereas women preferred black, gray, and white. Both genders valued the stretchiness, wicking, and breathability. Men prioritized durability, whereas women prioritized washability and sustainability. Men favored zippers, buckles, hooks, and loops for fastening, whereas women preferred zippers, hooks, loops, and no fasteners, but also considered snaps, tying cords, and micromotors. Therefore, it is recommended that MSCs for men be casual pants in gray, black, or blue, with preferred fasteners. For women, it can be a minimal-style innerwear in black, gray, or white with their favored fasteners.

### Differences in age

**Fig 7:**
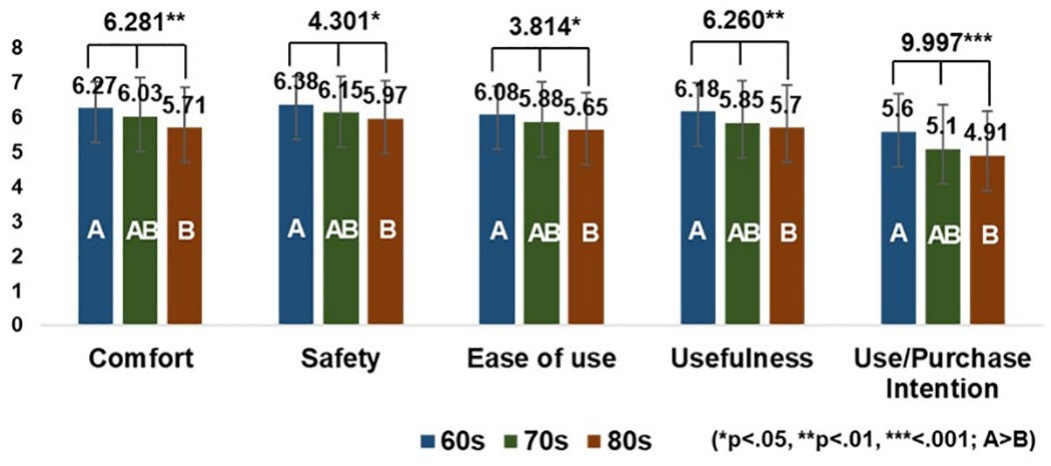
One-way ANOVA results in considering aspects.

Based on the one-way ANOVA tests, significant differences were found in the considerations for using and purchasing MSCs across age groups (Fig 7). Those in their 60s placed higher importance on comfort (*p* < .01), safety (*p* < .05), ease of use (*p* < .01), and usefulness (*p* < .01) compared to the other age groups. People in their 60s also had higher use and purchase intentions than those in their 70s and 80s, with those in their 80s considering the least. Therefore, while MSCs can initially target users in their 60s, developers should prioritize safety, comfort, usefulness, and ease of use.

**Fig 8:**
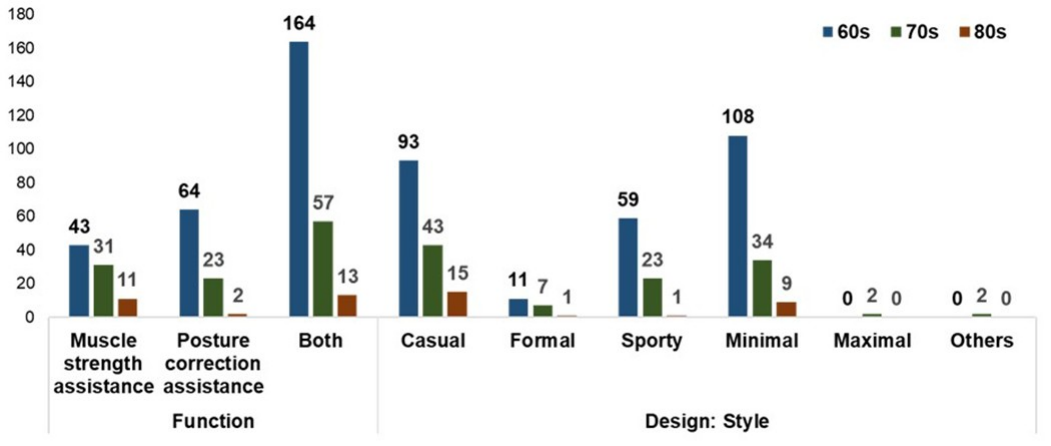
Results of chi-square test of age.

According to the results of Pearson’s chi-square test (expected frequency < 5; sample size > 40), there were significant differences among different ages in function and style (*p* < .05) (Fig 8). First, all age groups opted for both functions of posture correction assistance and muscle strength assistance, but those in their 60s wanted posture correction assistance more than muscle strength assistance, and those in their 70s and 80s wanted muscle strength assistance more. This may be because people in their 60s have greater muscle strength than those in other age groups. After 70 years, muscle mass loss accelerates further, resulting in a strength decline every ten years [12]. Thus, MSCs can be developed with both functions but with more emphasis on posture correction assistance for older adults in their 60s and muscle strength assistance for those in their 70s and older. Second, there was a significant difference in design styles among age groups (*p* < .05). The top two were minimal and causal styles, but more wanted minimal styles among people in their 60s and casual styles among people in their 70s and 80s. Hence, designs can be developed to include minimal styles for older adults in their 60s and casual styles for other age groups.

### Exploratory factor analysis and validity and reliability

The results of the EFA indicated that all the factors had an eigenvalue greater than one. The factors explained 74.27% of the total variance in the data with factor loadings ranging from .47 .92. The result of Confirmatory Factor Analysis showed that the measurement model had a good fit (*χ*^2^ = 676.76 with 288 *df*, *χ*^*2*^*/df* = 2.35, CFI = .97, NNFI = .96, IFI = .97, RMSEA = .058, and SRMR = .0343). The reliability and convergent validity of the constructs were assessed using composite/construct reliability and the average variance extracted (AVE). All items loaded on the intended constructs significantly at p-value < .001, and the standardized factor loadings of the items ranged from .71 to. 93. In this study, the composite reliability of each construct was assessed and ranged from .90.96. The AVE ranged from .67 to .79 (Table 1). The measurement model exhibited acceptable reliability and discriminant and convergent validity.

**Table 1:** Measurement model properties.

Measurement Items*	CFA Standardized Factor Loading	Construct Reliability^a^	AVE^b^
*Comfort*		.95	.67
COM1	.79		
COM2	.77		
COM3	.86		
COM4	.71		
COM5	.81		
COM6	.84		
COM7	.82		
COM8	.88		
COM9	.90		
*Safety*		.96	.79
SAF1	.88		
SAF2	.88		
SAF3	.90		
SAF4	.89		
SAF5	.89		
SAF6	.89		
*Ease of Use*		.90	.69
EOU1	.85		
EOU2	.88		
EOU3	.87		
EOU4	.71		
*Usefulness*		.92	.73
UFN1	.79		
UFN2	.89		
UFN3	.90		
UFN4	.84		
*Purchase/Use Intention*		.91	.78
PI1	.93		
PI2	.93		
PI3	.78		

^a^ Composite Reliability = (∑ standardized loading)^2^/(∑ standardized loading)^2^ + ∑ measurement error.

^b^ Variance Extracted = ∑ (standardized loading)^2^/ ∑ (standardized loading)^2^ + ∑ measurement error.

^c^ Items dropped from final analysis due to poor factor loadings.

* Anchored with 7-point Likert-scale descriptors, from 1 = “Strongly disagree” to 7 = “Strongly agree”.

The model proposed in this study was tested using structural equation modeling (SEM) with maximum likelihood estimation (Table 2). The structural model exhibited a good fit with the data (*χ*^2^ = 710.68 with 289 *df*, *χ*^2^/*df* = 2.46, CFI = 0.96, NNFI = 0.96, IFI = 0.96, RMSEA = 0.060, and SRMR = 0.035). Purchase or use intention was influenced by usefulness (*β* = 0.18, *t* = 2.52, *p* = .011) (H4) and the H4 was accepted. However, purchase or use intention was not influenced by comfort (*β* = 0.21, *t* = 2.08, *p* = .053) (H1), safety (*β* = 0.87, *t* = 13.02, *p* = .10) (H2), and ease of use (*β* = 0.22, *t* = 3.26, *p* = .72) (H3).

**Table 2:** Results: Structural model.

Endogenous Construct		SE^a^	t-value^b^
Purchase/Use Intention (R^2^ = .29)			
H1	Comfort	.53	1.94
H2	Safety	.30	-1.64
H3	Ease of Use	.35	-.37
H4	Usefulness	.18	2.54*
Fit Statistics			
N		408	
χ^2^ (*df*)		710.68 (289)	
CFI		.96	
NNFI		.96	
IFI		.96	
RMSEA		.060	
SRMR		.035	

^a^ SE, Standardized estimate.

^b^ *p < .05, **p < .01, ***p < .001.

Multiple-group SEM analysis was used for the invariance test to examine the robustness of the structural model across gender and perceived health conditions as moderators. The model fit difference from the comparison of gender indicated that the coefficients for the two groups were significantly different (*Δ χ*^2^ = 4.75, *Δ df* = 1, *p* = .03) (Tables 3 & 4). However, the model fit difference from the comparison of perceived healthy condition (healthy group versus unhealthy group) indicated that the coefficients for the two groups were not significantly different (*Δ χ*^2^ = .01, *Δ df* = 1, *p* = .93).

**Table 3:** Multiple-group structural model invariance test.

Groups	Model Description	*χ* ^2^	*df*	*Δχ* ^2^	*Δ df*	Sig. *p*	Model Invariance
Gender	Based model (free estimation)	1147.02	581	4.75	1	.03	No (variance)
	Model with equality constraint imposed	1151.77	582				
Perceived Healthy/Unhealthy Group	Based model (free estimation)	1170.91	581	.01	1	.93	Yes (Invariance)
	Model with equality constraint imposed	1170.92	582				

**Table 4:** Within-group structural coefficients and significance for hypotheses.

Endogenous Construct		Male		Female	
		SE^a^	t-value^b^	SE^a^	t-value^b^
Purchase/Use Intention		R^2^ = .46		R^2^ = .17	
H1	Comfort	.45	1.95	.45	1.95
H2	Safety	.27	-1.27	.27	-1.26
H3	Ease of Use	.32	-.42	.32	-4.22
H4	Usefulness	.19	3.49***	.19	1.71

^a^SE, Standardized estimate;

^b^*p < .05, **p < .01, ***p < .001.

### Design guidelines and considering aspects

**Fig 9:**
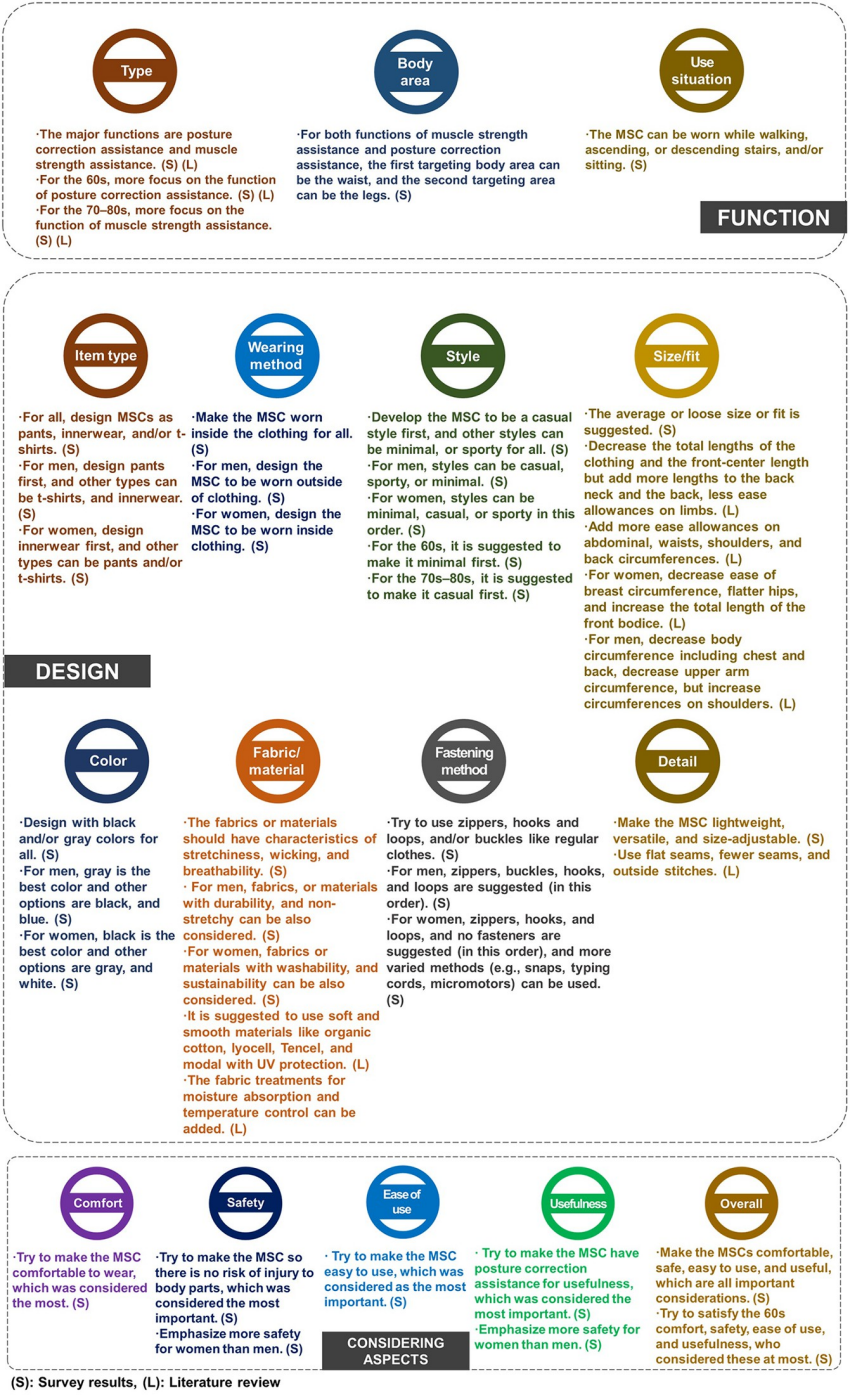
Design guidelines of MSCs.

Based on the results, the following design guidelines were derived in terms of function, design, aspects considered, and use/purchase intention for MSCs, as shown in Fig 9. The guidelines can be helpful for product designers and researchers when developing MSCs, considering what people need and want and what they consider when they use and purchase them. It is expected that these guidelines can pave the way for satisfying users’ needs and wants. To enhance the use and purchase intention, try to develop the MSC to be useful to increase use and purchase intention, considering the hypothesis. Also, men who showed higher use and purchase intention can be targeted first and then women.

## Conclusions

In conclusion, this study delved into the critical areas of MSCs for older adults, aiming to understand their considerations of aspects, needs, and wants, and to define design guidelines based on the results. These findings highlight the importance of designing MSCs that assist in muscle strength and posture correction, particularly during activities such as walking, climbing stairs, and sitting. These MSCs must be designed with comfort, safety, ease of use, and useful features, such as stretchy, breathable fabrics and convenient fasteners, to ensure a pleasant user experience. The preferences of men and women for MSCs were also explored, offering insights into varying design considerations based on gender and age.

This study emphasizes the crucial role of usefulness in influencing the use and purchase intentions of MSC among older adults. Developers need to prioritize these aspects to promote the adoption of innovative solutions. By adhering to design guidelines and considering the identified aspects, developers can create products that truly enhance the lives of older adults, promote independence, and contribute to the advancement of assistive technologies.

This research aimed to establish design guidelines catering to the needs, wants, and considerations of aspects, thereby contributing to the development of wearable solutions for addressing musculoskeletal issues in older adults.

This research serves as a foundational step toward meeting the evolving needs of an aging population and fostering innovation in MSCs. A more heterogeneous group with different demographic backgrounds needs to be examined. Future research should be conducted on measurements and user testing with the developed MSCs based on these research results in older adults.

## Supporting information

S1 File (DOCX)
