# Supplementary material for: Design guidelines for movement-assistive clothing based on a comprehensive understanding of older adults’ needs and preferences
Source: PLoS One. 2024 Mar 20;19(3):e0299434. doi: 10.1371/journal.pone.0299434 (PMC10954188; doi:10.1371/journal.pone.0299434)
Supplement: S1 File — (DOCX) [file pone.0299434.s001.docx]

**Data for the Fig 2. The needs and wants of older adults in functions for MSCs**

| Category | Sub-category | No. | % |
| --- | --- | --- | --- |
| Function | Both | 234 | 57.40 |
|  | Posture correction assistance | 89 | 21.80 |
|  | Muscle strength assistance | 85 | 20.80 |
| Body area for  muscle strength  assistance | Waist | 193 | 47.30 |
|  | Leg | 143 | 35.00 |
|  | Hip | 20 | 4.90 |
|  | Neck | 19 | 4.70 |
|  | Arm | 11 | 2.70 |
|  | Wrist | 10 | 2.50 |
|  | Ankle | 8 | 2.00 |
|  | Others | 4 | 1.00 |
|  | Hand | - | - |
|  | Foot | - | - |
| Body area for  posture correction  assistance | Waist | 251 | 61.50 |
|  | Leg | 63 | 15.40 |
|  | Hip | 21 | 5.10 |
|  | Others | 8 | 2.00 |
|  | Ankle | 6 | 1.50 |
|  | Foot | 3 | 0.70 |
|  | Wrist | 3 | 0.70 |
|  | Arm | 2 | 0.50 |
|  | Hand | - | - |
| Use situation | Walking | 206 | 50.50 |
|  | Ascending or descending stairs | 85 | 20.80 |
|  | Sitting | 58 | 14.20 |
|  | Standing | 28 | 6.90 |
|  | Holding objects | 24 | 5.90 |
|  | Others | 7 | 1.70 |

**Data for the Fig 3. The needs and wants of older adults in designs** **for MSCs**

| Category | Sub-category | No. | % |
| --- | --- | --- | --- |
| Item type | Pants | 160 | 39.20% |
|  | Innerwear | 119 | 29.20% |
|  | T-shirt | 66 | 16.20% |
|  | Outer | 32 | 7.80% |
|  | Dress/bodysuit | 11 | 2.70% |
|  | Shirt/blouse | 10 | 2.50% |
|  | Accessory | 5 | 1.20% |
|  | Skirt | 3 | 0.70% |
|  | Jewelry | 2 | 0.50% |
| Wearing method | Inside of the clothing | 280 | 68.60% |
|  | Outside of the clothing | 128 | 31.40% |
| Style | Casual | 151 | 37.00% |
|  | Minimal | 151 | 37.00% |
|  | Sporty | 83 | 20.30% |
|  | Formal | 19 | 4.70% |
|  | Maximal | 2 | 0.50% |
|  | Others | 2 | 0.50% |
| Size/fit | Average | 218 | 53.40% |
|  | Loose | 159 | 39.00% |
|  | Tight | 23 | 5.60% |
|  | Very loose | 8 | 2.00% |
|  | Very tight | 0 | 0.00% |
| Color | Black | 115 | 28.20% |
|  | Gray | 100 | 24.50% |
|  | White | 55 | 13.50% |
|  | Blue | 51 | 12.50% |
|  | Purple | 36 | 8.80% |
|  | Green | 19 | 4.70% |
|  | Orange | 13 | 3.20% |
|  | Others | 9 | 2.20% |
|  | Red | 7 | 1.70% |
|  | Yellow | 3 | 0.70% |
| Fabric/material | Stretchiness | 121 | 29.70% |
|  | Wicking | 92 | 22.50% |
|  | Breathability | 88 | 21.60% |
|  | Washability | 45 | 11.00% |
|  | Durability | 30 | 7.40% |
|  | Sustainability | 24 | 5.90% |
|  | Non-stretchiness | 7 | 1.70% |
|  | Anti-stain | 1 | 0.20% |
| Fastening method | Zipper | 124 | 30.40% |
|  | Hooks and loops | 104 | 25.50% |
|  | Buckle | 81 | 19.90% |
|  | Button | 36 | 8.80% |
|  | None | 26 | 6.40% |
|  | Snap | 19 | 4.70% |
|  | Tying cords | 13 | 3.20% |
|  | Micromotor (e.g., Boa motor) | 4 | 1.00% |
|  | Others | 1 | 0.20% |
| Detail | Lightweight | 147 | 36.00% |
|  | Versatility | 94 | 23.00% |
|  | Size-adjustability | 91 | 22.30% |
|  | Cellphone connection | 40 | 9.80% |
|  | Modular design | 25 | 6.10% |
|  | Thinness | 11 | 2.70% |

**Data for the Fig 4. Considering aspects when purchasing MSCs**

| Category | | M | SD | % above 4 |
| --- | --- | --- | --- | --- |
| Comfort | Overall comfort | 6.36 | 0.95 | 100.0 |
|  | Movement comfort | 6.29 | 1.02 | 99.0 |
|  | Static comfort | 6.25 | 1.05 | 97.8 |
|  | Wear comfort | 6.19 | 1.04 | 98.8 |
|  | No tiresome | 6.17 | 1.1 | 96.8 |
|  | Sound comfort | 6.13 | 1.16 | 95.8 |
|  | Stability | 6.13 | 1.1 | 96.8 |
|  | Thermal comfort | 6.10 | 1.08 | 98.3 |
|  | Pressure comfort | 5.88 | 1.23 | 94.4 |
| Safety | No risk of injury to body parts | 6.37 | 1.02 | 99.8 |
|  | Safety when using its features | 6.32 | 0.99 | 98.0 |
|  | Safe electrical and clothing components | 6.30 | 1.00 | 98.5 |
|  | Safe when wearing | 6.29 | 0.95 | 97.5 |
|  | No body issues after the use | 6.28 | 0.98 | 99 |
|  | Reaction to emergency (e.g., condition abnormalities or equipment malfunctions) | 6.19 | 1.09 | 96.8 |
| Ease of use | Ease of using functions | 6.14 | 1.07 | 96.6 |
|  | Ease of wear | 6.12 | 1.05 | 97.8 |
|  | Ease of understanding how to use | 6.09 | 1.04 | 97.3 |
|  | Can wear it quickly | 5.66 | 1.17 | 95.1 |
| Usefulness | Posture correction assistance | 6.19 | 1.07 | 96.8 |
|  | Muscle strength assistance | 6.14 | 1.10 | 98.0 |
|  | Enhance movements | 5.99 | 1.15 | 95.6 |
|  | Provides lots of benefits | 5.91 | 1.10 | 96.3 |
| Use/purchase intention | Benefits | 5.56 | 1.21 | 95.8 |
|  | Use/wear intention | 5.40 | 1.32 | 90.9 |
|  | Purchase intention | 5.29 | 1.31 | 90.4 |

**Data for the Fig 5. Results of t-tests of gender (mean, t-value, and p-value)**

| Category | Gender | | *t* |
| --- | --- | --- | --- |
|  | Men (*N*=204) | Women (*N* =204) |  |
| Comfort | 6.09 | 6.25 | -1.79 |
| Safety | 6.18 | 6.40 | -2.51* |
| Ease of use | 5.92 | 6.09 | -1.81 |
| Usefulness | 5.91 | 6.20 | -3.03** |
| Use/purchase intention | 5.53 | 5.31 | 1.85 |

**p*<.05, ***p*<.01

**Data for the Fig 6. Results of Chi-square test of gender**

| Needs and wants in function | | Male | Female | Pearson Chi-Square  Asymptotic Sig. (two-tailed) |
| --- | --- | --- | --- | --- |
| Needs and wants in designs | | Male | Female | Pearson Chi-Square  Asymptotic Sig. (two-tailed) |
| Item type | T-shirt | 46 | 20 | .000 |
|  | Pants | 96 | 64 |  |
|  | Innerwear | 38 | 81 |  |
|  | Outer | 22 | 10 |  |
|  | One-piece | - | 11 |  |
|  | Skirt | - | 3 |  |
|  | Shirt/blouse | 1 | 9 |  |
|  | Accessory | 1 | 4 |  |
|  | Jewelry | - | 2 |  |
|  | Total | 204 | 204 |  |
| Wearing method | Inside of the clothing | 124 | 156 | .001 |
|  | Outside of the clothing | 80 | 48 |  |
|  | Total | 204 | 204 |  |
| Style | Casual | 90 | 61 | .000 |
|  | Formal | 8 | 11 |  |
|  | Sporty | 56 | 27 |  |
|  | Minimal | 48 | 103 |  |
|  | Maximal | 1 | 1 |  |
|  | Others | 1 | 1 |  |
|  | Total | 204 | 204 |  |
| Color | Black | 51 | 64 | .000 |
|  | Gray | 61 | 39 |  |
|  | White | 21 | 34 |  |
|  | Blue | 40 | 11 |  |
|  | Purple | 13 | 23 |  |
|  | Green | 10 | 9 |  |
|  | Yellow | - | 3 |  |
|  | Orange | 6 | 7 |  |
|  | Red | 2 | 5 |  |
|  | Others | - | 9 |  |
|  | Total | 204 | 204 |  |
| Fabric/material | Non-stretchiness | 4 | 3 | .006 |
|  | Stretchiness | 66 | 55 |  |
|  | Wicking | 49 | 43 |  |
|  | Breathability | 47 | 41 |  |
|  | Durability | 18 | 12 |  |
|  | Washability | 15 | 30 |  |
|  | Anti-stain | 1 | - |  |
|  | Sustainability | 4 | 20 |  |
|  | Total | 204 | 204 |  |
| Fastening method | Buckle | 57 | 24 | .000 |
|  | Button | 19 | 17 |  |
|  | Hooks and loops | 48 | 56 |  |
|  | Zipper | 63 | 61 |  |
|  | Micromotor | 1 | 3 |  |
|  | Snap | 4 | 15 |  |
|  | Tying cords | 5 | 8 |  |
|  | None | 7 | 19 |  |
|  | Others | - | 1 |  |
|  | Total | 204 | 204 |  |

Expected frequency < 5

**Data for the Fig 7. One-way ANOVA results in considering aspects**

| Age | 60s (n=271) | 70s (n=111) | 80s (n=26) | F |
| --- | --- | --- | --- | --- |
|  | M (SD) | M (SD) | M (SD) |  |
| Comfort | 6.27 (0.77) ^A^ | 6.03 (1.11) ^AB^ | 5.71 (1.16) ^B^ | 6.281**** |
| Safety | 6.38 (0.82) ^A^ | 6.15 (1.04) ^AB^ | 5.97 (1.08) ^B^ | 4.301*** |
| Ease of use | 6.08 (0.83) ^A^ | 5.88 (1.14) ^AB^ | 5.65 (1.07) ^B^ | 3.814*** |
| Usefulness | 6.18 (0.82) ^A^ | 5.85 (1.21) ^AB^ | 5.70 (1.25) ^B^ | 6.26**** |
| Use/Purchase Intention | 5.60 (1.10) ^A^ | 5.10 (1.27) ^AB^ | 4.91 (1.27) ^B^ | 9.997***** |

**p*<.05, ***p*<.01, ****p*<.001; Scheffe test results: A>B

**Data for the Fig 8. Results of Chi-square test of age**

| Function and design | | 60s | 70s | 80s | Pearson Chi-Square  Asymptotic Sig. (two-tailed) |
| --- | --- | --- | --- | --- | --- |
| Function | Muscle strength assistance | 43(15.87%) | 31(27.93%) | 11(42.31%) | .003 |
|  | Posture correction assistance | 64(23.62%) | 23(20.72%) | 2(7.69%) |  |
|  | Both | 164(60.52%) | 57(51.35%) | 13(50%) |  |
|  | Total | 271 | 111 | 26 |  |
| Design: style | Casual | 93(34.31%) | 43(38.74%) | 15(57.69%) | .022 |
|  | Formal | 11(4.05%) | 7(6.31%) | 1(3.85%) |  |
|  | Sporty | 59(21.77%) | 23(20.72%) | 1(3.85%) |  |
|  | Minimal | 108(39.85%) | 34(30.63%) | 9(34.62%) |  |
|  | Maximal | - | 2(1.80%) | - |  |
|  | Others | - | 2(1.80%) | - |  |
|  | Total | 271 | 111 | 26 |  |

Expected frequency < 5

**A table for the Fig 9. Design guidelines of MSCS**

| Category | | Design guidelines |
| --- | --- | --- |
| Function | Type | ·The major functions are posture correction assistance and muscle strength assistance. (S) (L)  ·For the 60s, more focus on the function of posture correction assistance. (S) (L)  ·For the 70–80s, more focus on the function of muscle strength assistance. (S) (L) |
|  | Body areas | ·For both functions of muscle strength assistance and posture correction assistance, the first targeting body area can be the waist, and the second targeting area can be the legs. (S) |
|  | Use situation | ·The MSC can be worn while walking, ascending, or descending stairs, and/or sitting. (S) |
| Design | Item type | ·For all, design MSCs as pants, innerwear, and/or t-shirts. (S)  ·For men, design pants first, and other types can be t-shirts, and innerwear. (S)  ·For women, design innerwear first, and other types can be pants and/or t-shirts. (S) |
|  | Wearing method | ·Make the MSC worn inside the clothing for all. (S)  ·For men, design the MSC to be worn outside of clothing. (S)  ·For women, design the MSC to be worn inside clothing. (S) |
|  | Style | ·Develop the MSC to be a casual style first, and other styles can be minimal, or sporty for all. (S)  ·For men, styles can be casual, sporty, or minimal in this order. (S)  ·For women, styles can be minimal, casual, or sporty in this order. (S)  ·For the 60s, it is suggested to make it minimal first. (S)  ·For the 70s–80s, it is suggested to make it casual first. (S) |
|  | Size/fit | ·The average or loose size or fit is suggested. (S)  ·Decrease the total lengths of the clothing and the front-center length but add more lengths to the back neck and the back, less ease allowances on limbs. (L)  ·Add more ease allowances on abdominal, waists, shoulders, and back circumferences. (L)  ·For women, decrease ease of breast circumference, flatter hips, and increase the total length of the front bodice. (L)  ·For men, decrease body circumference including chest and back, decrease upper arm circumference, but increase circumferences on shoulders. (L) |
|  | Color | ·Design with black and/or gray colors for all. (S)  ·For men, gray is the best color and other options are black, and blue. (S)  ·For women, black is the best color and other options are gray, and white. (S) |
|  | Fabric/ material | ·The fabrics or materials should have characteristics of stretchiness, wicking, and breathability. (S)  · For men, fabrics, or materials with durability, and non-stretchy can be also considered. (S)  ·For women, fabrics or materials with washability, and sustainability can be also considered. (S)  ·It is suggested to use soft and smooth materials like organic cotton, lyocell, Tencel, and modal with UV protection. (L)  ·The fabric treatments for moisture absorption and temperature control can be added. (L) |
|  | Fastening method | ·Try to use zippers, hooks and loops, and/or buckles like regular clothes. (S)  ·For men, zippers, buckles, hooks, and loops are suggested (in this order). (S)  ·For women, zippers, hooks, and loops, and no fasteners are suggested (in this order), and more varied methods (e.g., snaps, typing cords, micromotors) can be used. (S) |
|  | Detail | ·Make the MSC lightweight, versatile, and size-adjustable. (S)  ·Use flat seams, fewer seams, and outside stitches. (L) |
| Considering aspects | Comfort | · Try to make the MSC comfortable to wear, which was considered the most. (S) |
|  | Safety | ·Try to make the MSC so there is no risk of injury to body parts, which was considered the most important. (S)  ·Emphasize more safety for women than men. (S) |
|  | Ease of use | · Try to make the MSC easy to use, which was considered as the most important. (S) |
|  | Usefulness | · Try to make the MSC have posture correction assistance for usefulness, which was considered the most important. (S)  ·Emphasize more safety for women than men. (S) |
|  | Overall | ·Make the MSCs comfortable, safe, easy to use, and useful, which are all important considerations. (S)  ·Try to satisfy the 60s comfort, safety, ease of use, and usefulness, who considered these at most. (S) |
| Use/purchase intention |  | ·Try to develop the MSC to be useful to increase use and purchase intention. (S)  ·Men who showed higher use and purchase intention can be targeted first and then women. (S) |

(S): Survey results, (L): Literature review
